# Rat Merkel Cells Are Mechanoreceptors and Osmoreceptors

**DOI:** 10.1371/journal.pone.0007759

**Published:** 2009-11-09

**Authors:** Nicholas Boulais, Jean-Pierre Pennec, Nicolas Lebonvallet, Ulysse Pereira, Nathalie Rougier, Germaine Dorange, Christophe Chesné, Laurent Misery

**Affiliations:** 1 University of Brest, European University of Brittany, Laboratory on Nervous Factors and Tissular Structure, EA4326, CHU, Brest, France; 2 Bioprédic International, Rennes, France; Tufts University, United States of America

## Abstract

Merkel cells (MCs) associated with nerve terminals constitute MC-neurite complexes, which are involved in slowly-adapting type I mechanoreception. Although MCs are known to express voltage-gated Ca^2+^ channels and hypotonic-induced membrane deformation is known to lead to Ca^2+^ transients, whether MCs initiate mechanotransduction is currently unknown. To answer to this question, rat MCs were transfected with a reporter vector, which enabled their identification. Their properties were investigated through electrophysiological studies. Voltage-gated K^+^, Ca^2+^ and Ca^2+^-activated K^+^ (K_Ca_) channels were identified, as previously described. Here, we also report the activation of Ca^2+^ channels by histamine and their inhibition by acetylcholine. As a major finding, we demonstrated that direct mechanical stimulations induced strong inward Ca^2+^ currents in MCs. Depolarizations were dependent on the strength and the length of the stimulation. Moreover, touch-evoked currents were inhibited by the stretch channel antagonist gadolinium. These data confirm the mechanotransduction capabilities of MCs. Furthermore, we found that activation of the osmoreceptor TRPV4 in FM1-43-labeled MCs provoked neurosecretory granule exocytosis. Since FM1-43 blocks mechanosensory channels, this suggests that hypo-osmolarity activates MCs in the absence of mechanotransduction. Thus, mechanotransduction and osmoreception are likely distinct pathways.

## Introduction

The sense of touch is not fully understood in mammals [1]. The slowly adapting type I mechanoreceptor (SAI) formed by the Merkel cell (MC)-neurite complex is critical for shape and texture discrimination [2]. SAI is concentrated at touch sensitive areas of the skin, such as fingertips, lips, touch domes and vibrissal outer root sheath in rodents (for review see [3], [4]). However, since previous work has produced conflicting results, it is still unclear whether MCs are able to initiate mechanotransduction by themselves [5], [6]. Mechanotransduction requires stimulation of mechanically sensitive proteins, the opening of ion channels and the subsequent activation of nerve terminals, which generate action potentials. For MCs, electrophysiological evidence has demonstrated the presence of L-type (Ca_v_1.2), P/Q-type (Ca_v_2.1) and N-type (Ca_v_2.2) voltage-gated Ca^2+^ channels and the role of Ca^2+^-induced Ca^2+^ release (CICR) in the evocation of robust intracellular Ca^2+^ transients [7], [8], [9]. Consecutive synaptic transmission to somatosensory neurons was bolstered by tight connections with nerve terminals, which were observed by confocal imaging and ultrastructural studies [10], [11]. Furthermore, essential components of the synaptic machinery were detected [12], [13], [14]. However, direct mechanical stimulation previously failed to activate quinacrine-labeled MCs [7].

Fluorescent dyes like quinacrine or FM1–43 were successfully used to identify MCs in epidermal cell cultures [15]. Unfortunately, quinacrine inhibits some ion channels and Ca^2+^ uptake in neuroendocrine cells [16], [17]. FM1–43 is a useful tool for studying neuropeptide secretion and membrane trafficking [18]. It was also found to be an efficient blocker of mechanosensory ion channels in sensory cells, such as neurons and hair cells [19], [20]. Therefore, although these dyes specifically label MCs in epidermal cell cultures, their biological effects have to be considered. Hence, a remaining challenge is the identification of rare functional MCs among predominant keratinocytes.

To overcome this problem, Lumpkin *et al*. generated transgenic mice in which the enhancer of the neural transcription factor mouse atonal homolog1 (Math1) drove the expression of GFP [21]. In the epidermis, Math1 is specifically expressed by MCs. Fluorescence-activated cell sorting enabled isolation of a population of cells constituted by 85 to 95% MCs. Evaluation of this purified cell population indicated that MCs express presynaptic proteins [12] and that hypotonic-induced membrane deformation initiated Ca^2+^ signaling [22] modulated by Ca^2+^-activated K^+^ (BK_Ca_) channels [23].

As MCs express the osmoreceptor TRPV4 (transient receptor potential vanilloid type 4) [22], [24] and because its activation by hypotonic solution or the phorbol derivative 4αPDD (4α-phorbol-12, 13-didecanoate) also leads to Ca^2+^ influx [25], [26], [27], we addressed the hypothesis that different pathways can be initiated in hypotonic-stimulated MCs.

Based on the strategy used by Lumpkin *et al*., we identified the enhancer of Math1 in the rat genome by sequence alignment. This rat sequence was fused into a β-galactosidase expression vector system. Epidermal cells from rat footpads were isolated and then transfected with the engineered vector in order to permit identification of functional MCs. To determine whether MCs respond to both mechanical and osmotic stimuli, we electrophysiologically analyzed direct mechanical stimulations of MCs in an osmotic medium. We then used hypotonic solutions or 4αPDD to stimulate MCs in the absence of mechanotransduction. For this last experiment, we labeled MCs with the mechanosensory channel inhibitor FM1–43, which allowed us to follow membrane trafficking. Our electrophysiological recordings confirmed previous data on MCs: they express Ca^2+^, K^+^ and Ca^2+^-activated K^+^ channels. In addition, we found that MCs responded to histamine and acetylcholine (ACh). More interestingly, we demonstrated that MCs express stretch-channels capable of transducing the strength and the length of a mechanical stimulation. We also showed that hypotonicity or exposure to 4αPDD was sufficient to induce neurosecretory granule exocytosis from FM1–43-labelled MCs. Taken together, these results confirm mechanotransduction properties of MCs and support the hypothesis that dense-core granule exocytosis is linked to events other than touch, such as hypotonicity and TRPV4 activation. To conclude, we hypothesize that mechanoreception and osmoreception differentially activate MCs. Mechanoreception may initiate synaptic release while osmoreception could induce dense-core granule exocytosis.

## Results

### Transfection of Merkel Cells

**Figure 1:**
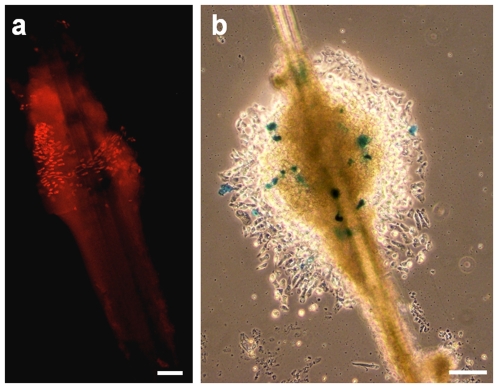
Transfection of MCs from rat vibrissae. MCs are densely represented in the upper part of the vibrissal outer root sheath as demonstrated by CK20 immunostaining (a). The expression pattern of the neural transcription factor Math1 was restricted to this area, as demonstrated by the read out of the reporter vector pMath1-β-galactosidase (b). Scale bars: 50 µm.

In order to define a suitable transfection protocol to transiently transfect MCs, the first tests were carried out on whiskers. This model appeared more appropriate than footpads because MCs are more numerous in whiskers and they are located specifically at the upper part of the outer root sheath, as demonstrated by anti-cytokeratin 20 immunostaining (Figure 1a). This localization proper to MCs allowed us to assess the suitability of our vector, because coupled β-galactosidase labeling and immunostaining was very difficult. In our experimental conditions, the best results were obtained with Tranfast. Using this reagent, most Math1-driven β-galactosidase-expressing cells were observed at the expected location (Figure 1b). We failed to detect transfected MCs cells following transfection by DEAE-dextran/chloroquine, Lipofectine or Nanofectine. A low number of β-galactosidase-expressing cells were observed when we used Lipofectamine LTX or PEI. Unfortunately, as the number of MCs per whisker varied considerably, the yield of transfected cells compared to the total number of MCs could not be assessed.

**Figure 2:**
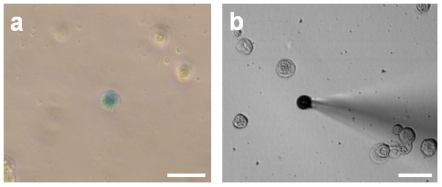
Identification of transfected MCs from rat footpads for electrophysiological analyses. The epidermis of a rat footpad was stripped off of the dermis and the cells were dissociated and transfected with pMath1-β-galactosidase in order to permit detection of MCs (a). Transfected MCs represented 1.35% (+/−0.73) of the epidermal cells. Electrophysiological recordings targeting blue cells were performed in the cell-attached macro-patch configuration (b). Scale bars: 20 µm.

As electrophysiological studies required isolated cells, epidermal cells from rat footpads were preferentially used. Basal and suprabasal epidermal cells were dissociated as described in the Materials and Methods section. Immunofluorescence analyses against CK20 showed that MCs usually represent 2.37±1.67% of the cultured epidermal cells (n = 3). Retrieved cells were transfected with pMath1-β-galactosidase using Transfast. β-Galactosidase staining enabled us to distinguish MCs from other cell types (Figure 2). Briefly, 1.35±0.73% (n = 3) of the dissociated epidermal cells appeared blue, indicating that an average of 57% of MCs were transfected by this approach.

### MCs Possess Voltage-Activated Ca^2+^ Channels

**Figure 3:**
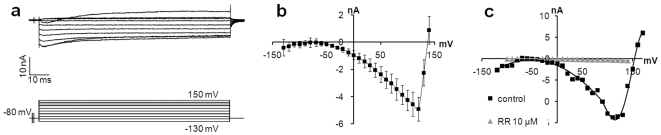
Voltage-activated Ca^2+^ channel are produced by MCs. A majority of MCs expressed Ca^2+^ currents. (a) Representative inward Ca^2+^ currents from transfected MCs in response to a series of depolarizing pulses. (b) Average of steady-state current densities during the depolarizing step, plotted as a function of membrane potential. The reverse potential at approximately 150 mV is representative of Ca^2+^ current (n = 11, error bars are s.e.m.). (c) RR at 10 µM (grey triangles) inhibited inward currents observed in control conditions (black squares), which confirmed the presence of voltage-activated Ca^2+^ channels.

MCs may use voltage-activated Ca_V_2.1 (P/Q-type), Ca_V_2.2 (N-type) and Ca_V_1.2 (L-type) Ca^2+^ channels to produce Ca^2+^ transients through the release of internal Ca^2+^ stores. BK_Ca_ channels, for which α (KCNMA1) and β subunits (KCNMB1, KCNMB2, KCNMB4) are expressed in MCs, are thought to restore the membrane potential [23]. In this study, we recorded membrane currents of β-galactosidase-expressing cells in response to 100-ms voltage steps from −120 mV to 150 mV. Recordings were performed in the cell-attached macro-patch configuration in an osmotic saline buffer. In most cases (33/50), inward currents activated by application of depolarizing pulses were evidenced. Under our conditions, long lasting currents showed no fast inactivation, started to activate at −50 mV, reached a peak at 100 mV, exhibited a reversal potential of about +140 mV and a maximal conductance of 280 nS according to the mean current-voltage relationship (Figure 3a; b, n = 11, error bars denote s.e.m.). According to the Nernst equation, we found an average intracellular Ca^2+^ concentration of 70 nM. The peaks of inward currents varied, depending on the number of channels that were recorded at the same time. The activation kinetics and the permeation properties are consistent with L-type Ca^2+^ channels. After a 3-min incubation with 10 µM Ruthenium red (RR), an inhibitor of Ca^2+^ channels, inward currents were fully inhibited, which suggested that inward currents were mainly carried by Ca^2+^ channels (Figure 3c). Ca^2+^ channels usually induce signal transmission and depolarization through Ca^2+^ transients in excitatory cells. They are also known to trigger the CICR pathway in MCs [9].

### MCs Produce Voltage Activated K^+^ Channels

**Figure 4:**
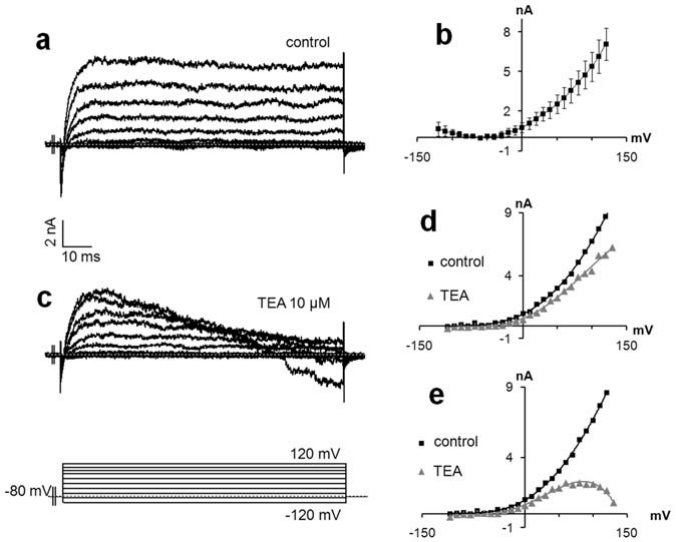
MCs produced voltage-activated K^+^ channels. (a) A representative slow activated outward K^+^ current lacking an inactivating component. (b) Average steady-state current densities during depolarizing pulses, plotted as a function of voltage (n = 7, error bars denote s.e.m.). (c) TEA (10 mM), a classical inhibitor of K^+^ channels, decreased current densities compared to control conditions. (a) Non-inactivated currents and slow-activated currents with an inactivating component were observed. Peak (d) and steady-state from the last 40 ms. (e) Current densities during the depolarizing step were plotted as a function of membrane potential for control conditions (black squares) or with TEA (grey triangles). (d) The decreased current densities demonstrated the presence of K^+^ channels. (e) Steady-state current densities highlighted the inactivating component starting around 80 mV.

Outward currents were detected in 34% (17/50) of the recorded MCs. These currents were generally (14/17) delayed slow activated K^+^ currents with no spontaneous inactivation. They usually began to activate at −40 mV and reached a maximal activation state at +80 mV for an average conductance of 60 nS (Figure 4a, b; n = 7). Tetraethylammonium (TEA), a classical inhibitor of voltage-activated K^+^ channels, was added to the medium at a final concentration of 10 mM and records were compared to control conditions. Without TEA, we detected non-inactivated outward currents, which activated at −40 mV and reached a maximum activation state at +80 mV for a conductance of 90 nS. Three min after the addition of TEA, we observed non-inactivated outward K^+^ currents activated at −30 mV with a maximum activation state at +40 mV for a conductance of 50 nS. The amplitude of the currents at +120 mV was decreased to 35% (from 8.7 to 5.6 nA) (Figure 4c, d). We also detected slow activated outward currents with maximum activation states at +20 mV for a conductance of 48 nS and an inactivating component that turned on at 80 mV, as demonstrated by the tail current at the steady state (Figure 4c, e).

**Figure 5:**
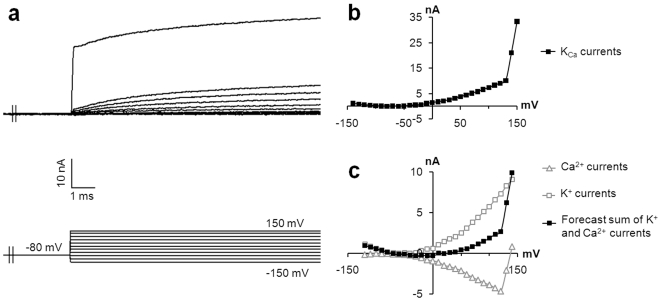
Ca^2+^-activated K^+^ channels in MCs. In three recordings, a sharp increase of outward current was detected, suggesting the presence of BK_Ca_ channels, which have already been described in MCs. (a) Representative voltage-dependent currents activated in response to a series of depolarizing pulses. (b) The current-voltage curve demonstrated a marked increase of the conductance around +130 mV, suggesting the involvement of different currents. (c) In order to determine whether these currents correspond to both Ca^2+^ and K^+^ currents, the two current-voltage curves from a previous recording were added. The pattern of the forecast current to voltage relationship (black squares) corresponded to the recorded currents.

In some records (3/17), outward currents with stronger amplitudes of approximately +130 to +150 mV were observed (Figure 5a, b). In these cases, the average conductance sharply increased from 115±36 nS to 900±190 nS (n = 3). The pattern of the current-voltage relationship indicated both K^+^ and Ca^2+^ currents, as indicated by merge modelling (Figure 5c). This increased intracellular Ca^2+^ concentration coupled to rectifying K^+^ channels suggested the involvement of previously described BK_Ca_ channels [23]. No voltage activated Na^+^ channels or fast inactivated Ca^2+^ channels (T type) were evidenced.

### Ca^2+^ Channels in MCs Are Modulated by Acetylcholine and Histamine

**Figure 6:**
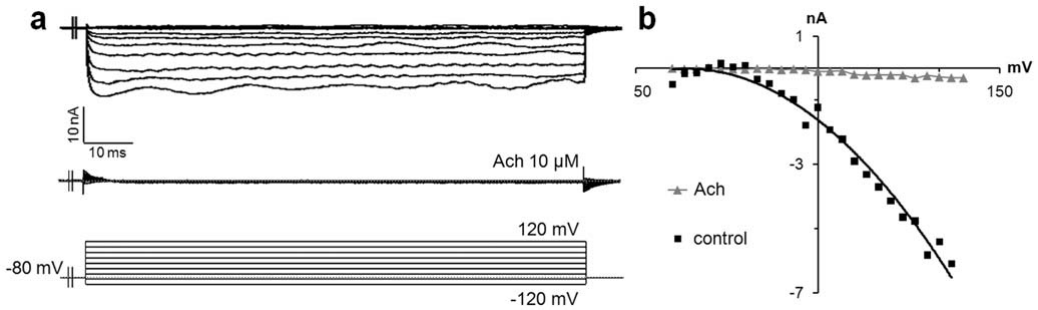
ACh inhibits inward currents in MCs. (a) Representative inward Ca^2+^ currents without an inactivating component (top traces) recorded in the control condition from transfected MCs of rat touch dome. These currents were almost entirely inhibited after two min of exposure to ACh (10 µM) (bottom traces). (b) Steady-state current densities were plotted as a function of voltage in normal conditions (black squares) and after stimulation with ACh (grey triangles) (n = 4).

ACh has pleiotropic roles in basic physiological functions. In the skin, ACh is known to regulate intracellular Ca^2+^ concentration via nicotinic (nAChR) and muscarinic (mAChR) receptors [28]. To investigate its effect on MCs, we added ACh (10 µM) to transfected MCs and measured Ca^2+^ currents after 2 min. In tested MCs, exposure to ACh almost entirely inhibited Ca^2+^ currents and led to a faint remaining inward current (Figure 6, n = 4). This inhibition suggested a signal transduction event through M2 or M4 mAChR [29].

**Figure 7:**
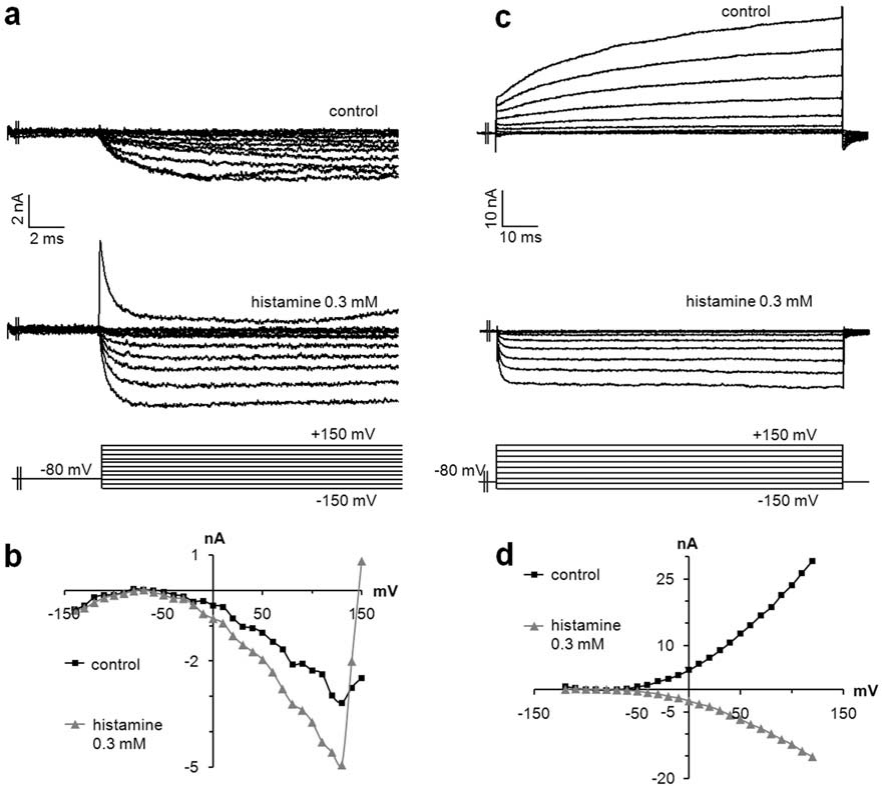
Histamine induces inward currents in MCs. The histamine H3 receptor was identified at the surface of MCs. Its activation induced inward Ca^2+^ currents in MCs. Representative current densities recorded during a series of depolarizing steps in control conditions (a, c: top traces). Inward slow activating Ca^2+^ currents (a) or outward fast activating K^+^ currents (b) were identified as revealed by the current-voltage relationships (b, d: black squares). In both conditions, the addition of histamine (300 µM) induced inward Ca^2+^ currents without inactivating components (a, c: bottom traces) as demonstrated by the current-voltage relationships (b, d: grey triangles).

Histamine H3 receptor (H3R) reduces inflammation and nociception. H3R has been recently described on MCs [30], but its function has not been defined in these cells. The addition of histamine (300 µM) induced inward Ca^2+^ currents in transfected MCs, whatever the currents found before (Figure 7). Histamine increased Ca^2+^ currents, not by modifying the voltage range of activation, but by significantly increasing the conductance by 80% (from 20 to 36 nS) and the peak current at +130 mV from 3.2 to 5 nA (Figure 7a, b). When an outward K^+^ current was observed, the addition of histamine at the same concentration provoked inversion of global currents, possibly through augmentation of inward Ca^2+^ currents and a decrease of outward currents (Figure 7c, d). Activation of histamine receptors on MCs induced depolarization through activation of Ca^2+^ channels, which suggests a potential regulatory role for these cells in skin inflammation.

### MCs Have Mechanosensory Stretch-Sensitive Channels

**Figure 8:**
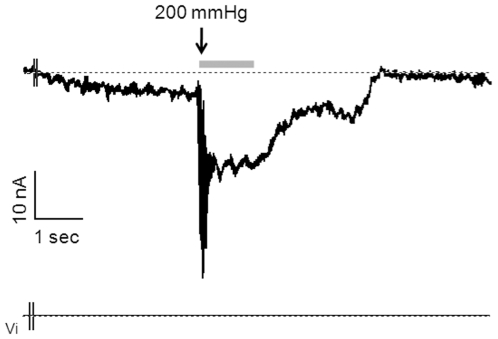
Suction initiates spontaneous inward currents in MCs. MCs are believed to function as a mechanoreceptors. To determine whether they respond to touch, suction (200 mmHg) was applied to transfected MCs from a rat footpad. Suction induced a strong, non-delayed, inward transient current. The decrease of inward currents at the second part of the recording is explained by the time required to stop the stimulation.

**Figure 9:**
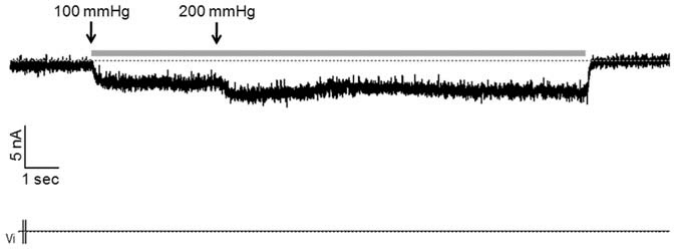
MCs transduce the strength and the length of the stimulation. A 100 mmHg-suction was applied to transfected MCs. Stimulation induced inward currents sustained throughout the stimulation (about 15 sec). An increase in the strength of the stimulation after 4 sec led to increased inward currents.

Previous investigations of the mechanosensory properties of MCs produced conflicting results [5], [31], [32]. Because direct mechanical stimulation failed to activate MCs [7], most authors used indirect hypo-osmotic stimulation to explore their mechanosensory properties [22], [27]. Here, we tested direct mechanical stimulation of MCs by applying controlled suctions through a patch pipette, which was monitored by a pressure gauge. A stimulation of 200 mmHg induced a strong transient depolarizing current in MCs. This inward current was not delayed (or delayed by less than 100 ms) and had amplitude of 40 nA. It was followed by a marked depolarizing sustained current lasting as long as the suction. The resting potential was restored at the end of the stimulation (Figure 8). We assessed membrane currents in MCs when mechanical stimulations were increased. MCs respond to a first stimulation of 100 mmHg by a stable depolarizing current of 2.2 nA, which was maintained during the four seconds of stimulation. The increase in pressure to 200 mmHg induced a much more significant depolarizing current of 3.5 nA, which was stable over the 11-second stimulation (Figure 9). Thus, MCs were able to give electrophysiological responses to mechanical stimulations performed over 15 seconds without accommodation and in a strength-dependent manner.

**Figure 10:**
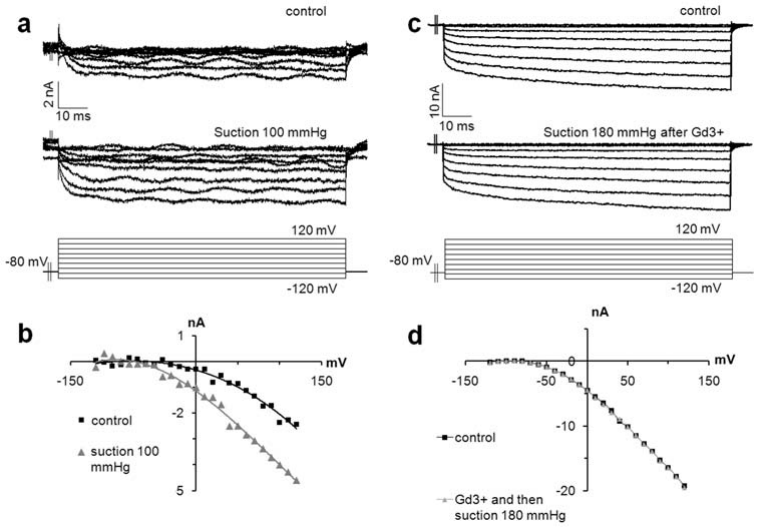
Touch-evoked inward currents are inhibited by Gadolinium. Gd^3+^ is a stretch-channel inhibitor. Once added to the medium (100 µM), Gd^3+^ inhibited inward currents induced by suction, supporting the presence of stretch channels in MCs. (a) Representative inward Ca^2+^ currents recorded in control conditions (top traces) and following the application of suction (bottom traces). (b) Current-voltage relationships demonstrating increased depolarizations during suction (grey triangles) compared to control conditions (black squares). (c) Recorded inward currents in control conditions (top traces) were not modified by suction when cells were pre-exposed to Gd^3+^ (bottom traces). (d) Steady-state current densities during the depolarizing step were plotted as a function of membrane potential.

Gadolinium(III) (Gd^3+^) is an inhibitor of non-selective cationic stretch channels. In order to confirm the presence of such channels on MCs, Gd^3+^ was applied at 100 µM. Voltage-dependant currents were recorded before and after mechanical stimulation. Before suction, slow inward currents were detected. When we applied a suction of −100 mmHg with an imposed potential, a marked augmentation of the current intensity was observed (+175±13%, n = 3). The current to voltage relationship revealed an increased inward conductance with permeability enhancement of 34.1±4.8% (Figure 10a, b). In the presence of Gd^3+^, suction up to 180 mmHg did not increase inward currents. In fact, inward currents were not modified to any degree. Therefore, Gd^3+^ inhibited the response to suction by blocking non-selective cationic stretch activated channels (Figure 10c, d). The current flowing through these channels should be essentially a Ca^2+^ current, considering the equilibrium potential of the current voltage relationship. Hence, MCs respond to deformation by producing slowly adapting Gd^3+^-sensitive mechanosensory channels that induced depolarization following activation.

### Hypotonic Stress and TRPV4 Activation Induce FM1-43-Loaded Neurosecretory Granules Exocytosis

**Figure 11:**
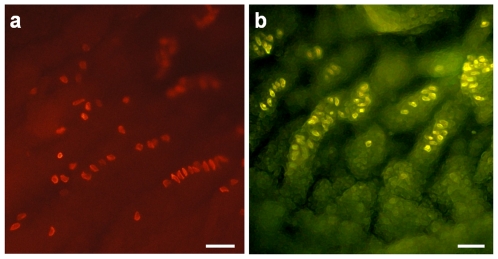
FM1–43 stained neurosecretory granules of MCs. FM1–43 is a fluorescent dye and a permeant blocker of mechanosensory channels, which stained the neurosecretory granules of neurons, hair cells, and MCs. (a) MCs are concentrated in rat footpads as demonstrated by CK20immunostaining. (b) Intraperitoneal injection of FM1–43 at 3 mg/kg in the rat allows for identification of MCs in the basal layer of the epidermis of rat footpads. Scale bars: 50 µm.

**Figure 12:**
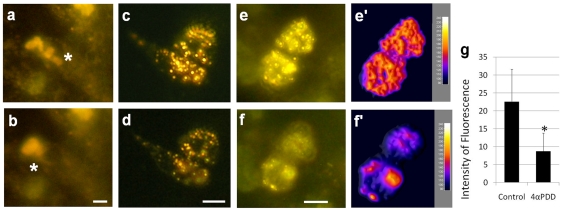
TRPV4 agonist induced FM1-43-loaded neurosecretory granule exocytosis. FM1–43 was injected intraperitoneally (3 mg/kg) in young rats. The following day, MCs from footpads were dissociated and cultured in isotonic medium (a, c, e). Exposure to hypotonic solution (b) or 4αPDD 1 µM (d, f), a TRPV4 agonist, induce neurosecretory granule exocytosis, as suggested by decreases in fluorescence intensity of 61% (n = 33) (g). Computational analyses showed movements of fluorescent particles from the middle to the periphery of the cells (e′, f′) which supported exocytosis. *P<0.01 significantly different from control. Scale bar in all pictures: 5 µm.

FM1–43 is a useful fluorescent dye for the monitoring of membrane trafficking in sensory cells. In addition, it inhibits mechanically-activated ion channels [18], [19], [20]. FM1–43 labels neurosecretory granules of MCs (Figure 11) [15]. In order to understand whether MCs react to hypo-osmolarity, rat FM1–43-labeled MCs were exposed to a hypotonic solution by the addition of water to the medium to a final osmolarity of 200 mosm. In this condition, we observed movement of neurosecretory granules from the cytoplasm to the plasma membrane and a decrease of intracellular fluorescence, which revealed neuropeptide exocytosis (Figure 12a, b). Because FM1–43 blocks mechanosensory function, this finding suggested that MCs can act as osmoreceptors.

Furthermore, we pharmacologically stimulated the osmoreceptor TRPV4 to avoid cell swelling. Analyses of TRPV4-deficient mice did not demonstrate a critical role for this receptor [22], but overlapping functions of the TRP channel have to be considered [33]. In the absence of mechanical stimulation, activation of TRPV4 by the specific agonist 4αPDD (1 µM) was sufficient to induce neurosecretory granule exocytosis, as demonstrated by the decreased fluorescence and the movement of granules to the membrane (Figure 12c–f). Computational analyses demonstrated changes in fluorescence intensity within cells after stimulation. Fluorescence in the middle of the cell waned (turned into blue) while peripheral fluorescence increased (turned into yellow-white) which show neurosecretory granules movement and probably exocytosis (Figure 12e′, f′). The global average fluorescence intensity in 4αPDD-stimulated MCs significantly (Student's paired t-test, *P*-value <0.01%) decreased of 61% (Figure 12g). Control stimulation with water had no effect in our experiments. Taken together, these results strongly suggest that MCs act as osmoreceptors in addition to acting as mechanoreceptors. Moreover, activation of TRPV4 appears sufficient to initiate dense-core granule exocytosis from MCs.

## Discussion

The mechanotransduction properties of MCs in the MC-neurite complex remain controversial [5], [6]. Recently, increasing evidence has demonstrated the importance of Ca^2+^ signaling to induce depolarization of MCs stimulated by hypo-osmolarity [9], [22], [23]. In this study, we described a process by which to transiently transfect rat MCs. By performing electrophysiological recordings, we report the presence of voltage–activated Ca^2+^, K^+^ and K_Ca_ channels in MCs. These findings confirm recently published data. Histamine and acetylcholine modulated the activation of Ca^2+^ channels in MCs. Importantly, we provide evidence that MCs act as mechanoreceptors because direct mechanical stimulation induced sustained strength-dependent depolarizations. The inhibition of these currents by Gd^3+^ confirmed the presence of cationic non-selective stretch activated channels on MCs. Finally, we demonstrated that hypo-osmolarity led to neurosecretory granule exocytosis, possibly through the activation of TRPV4 in FM1–43-labeled MCs. Taking into account that FM1–43 inhibits mechanotransducer channels [19], [20], our results suggest that MCs act as mechanoreceptors and osmoreceptors.

Recent reports extensively described the expression, production and activation of N-, P/Q- and L-type Ca^2+^ channels, K+ and K_Ca_ channels, as well as voltage-activated currents in MCs [7], [23]. In this work, similar currents were recorded and no Na^+^ current was identified, which confirmed the gathered electrophysiological data on MCs from touch domes, whisker follicles and footpads of rodents. Although Piskorowski et al. detected inward Ca^2+^ currents in half of the MCs (14/30) and after inhibition of masking K^+^ currents, in our model, we observed a majority of inward currents (33/50), while K^+^ and K_Ca_ currents were more sparse (14/50 and 3/50, respectively). The use of RR to inhibit Ca^2+^ channels generally did not allow disclosure of K^+^ currents in our experiments, which suggests that Ca^2+^ currents are not masking K^+^ currents. This finding is consistent with data for polarized cells and agrees with published immunostainings, which detected specific Ca^2+^ channels on microvilli or close to the nerve terminal. Conversely, K_Ca_ channels were found on the whole plasma membrane [23], but these channels were probably not activated when Ca^2+^ currents were blocked.

Ca^2+^ channels are involved in cell signalling, enzyme activation and neuropeptide release. Receptors to ACh had not yet been described on MCs. However, ACh is known to inhibit the Ca^2+^ current of N and P/Q type channels in neurons via the activation of presynaptic muscarinic M2 [34] and M4 receptors [29]. Moreover, ACh signalling often regulates neuropeptide release in neuroendocrine cells expressing VIP, like MCs [35], [36], through the activation of mAChR [37]. Hence, the expression of mAChR is highly probable on MCs. We provide electrophysiological evidence that AChRs are present on MCs and that their activation inhibits Ca^2+^ channels. As we also found that ACh inhibits VIP release in swine MC [36], this evidence provides a strong argument in favour of considering ACh as a modulator of MC secretory functions via mAChR. This finding also supports the involvement of N or P/Q type Ca^2+^ channels. No effect was observed on delayed K^+^ channels and no inward current was induced by ACh. These data suggest that ACh acts through muscarinic M2 receptors. However, a precise identification of the receptor type remains to be carried out. Furthermore, ACh appears to act as a neurotransmitter in MCs, similar to its role in some neuroendocrine cells that coproduce VIP and ACh [37] in the central nervous system.

The histamine H3 receptor was previously described at the surface of MCs [30]. This receptor mainly reduces neuromediator release in the brain [38] and peripherally inhibits inflammation and nociception [39]. In this study, we found that histamine induced the depolarization of rat MCs, rather than the inhibition of inward currents. We previously found that histamine increased VIP release from MC [36]. Therefore, we hypothesize that other activating histamine receptors are present in MCs. Otherwise, the H3 receptor may act differentially in MCs. Activation of MCs may be partly mediated by the release of histamine from cutaneous mast cells and because neuropeptides like VIP or CGRP, which are secreted by MCs, are known to modulate inflammatory processes, this finding might suggest putative immunomudulatory functions of MCs [35], [36]. This latter hypothesis is bolstered by the increased density in MCs observed in the context of inflammatory skin diseases [40], [41], [42], [43].

The SAI formed by the MC-neurite complex is critical for shape and texture discrimination [2]. However, despite evidence of synaptic capabilities [11], [12], successive analyses failed to confirm the mechanotransduction properties of MCs. Recently, it was demonstrated that cell swelling induced membrane deformation-initiated Ca^2+^ signaling in MCs. Unfortunately, this finding was not sufficient to establish the mechanotransduction properties of MCs because MCs express hypotonic-activated ion channels. Eleven receptors of the TRP superfamily were identified in MCs [22] and most of them react to several stimuli [33]. Moreover, a latency of 200 µsec was found for the SAI [44], while cell swelling activated MCs with a latency of 11 seconds. In our study, we applied mechanical stimuli by suction in iso-osmotic medium at neutral pH. Our results revealed touch-evoked inward currents that were not delayed. Observed depolarizing currents were strength-dependent and maintained throughout the stimulation without accommodation. Suction performed during voltage-gated current recordings allowed us to identify stretch channels on the current-voltage relationship, mostly carrying Ca^2+^. Finally, these currents were inhibited by Gd^3+^, a cationic non-selective stretch channel inhibitor. Taken together, these results firmly demonstrated that MCs are mechanoreceptors and strongly support the idea that MCs initiate mechanotransduction in the MC-neurite complex.

After establishing that MCs are mechanoreceptors, we tested whether MCs react to hypo-osmolarity without mechanical stimulation. Given that cell swelling induces membrane deformation, we labeled MCs with FM1–43, which is known to inhibit mechanotransducer channels. In addition, FM1–43 can be used to follow neurosecretory granule trafficking [18]. Here, we showed that hypo-osmolarity led to MC degranulation. This result links hypotonicity to neuroendocrine function. In addition, we stimulated the osmoreceptor TRPV4 by a pharmacological agonist to avoid cell swelling. TRPV4 is a cationic channel known to respond to hypo-osmolarity, acidification and membrane deformation [25], [45], [46], [47].

Although hypotonic-evoked inward currents were not impaired in TRPV4-null mice, we demonstrated here that activation of this receptor was sufficient to initiate neurosecretory granules movement (Figure 12). Since analyses revealed a decrease of 61% (n = 33) in fluorescence intensity, a release of neurosecretory granules is suggested. Therefore, TRPV4 activation must induce neuropeptides release. This result was confirmed by the increased amount of VIP released observed in 4α-PDD stimulated swine MC [36]. Thus, TRPV4 can be linked to osmoreception in MCs; however, other ion channels probably assume this function as well. Moreover, because Gd^3+^ was also found to inhibit TRPV4 [48], it can be hypothesized that TRPV4 also acts in mechanotransduction.

In summary, we have shown that MCs are mechanoreceptors and osmoreceptors. Mechanoreception involves Ca^2+^ signalling in MC and may be associated with synaptic release. Osmoreception is more related to neurosecretory functions with a longer latency than mechanoreception. Hence, these results support our recent study in swine MC in which we provide evidence of two distinct secretory pathways in regard to their Ca^2+^ dependency [36]. Furthermore, we demonstrated the participation of TRPV4 in osmoreception, although other ion channels are also likely to act as osmoreceptors. In addition, as Ca^2+^ channels are modulated by histamine and ACh, putative regulatory functions are likely in the cutaneous pathophysiological processes. Therefore, MCs fully belong to the somatosensory system, but still remain neuroendocrine cells that have a role in skin biology.

## Materials and Methods

### Animal Care

Experimentions were conducted in accordance with French government policies (Services véterinaires de la Santé et de la production animale, Ministère Français de l′Agriculture) and designed in accordance with recommendations of the regional ethical committee and the European Community directive no 86/609. Experimentations were permitted by departmental agreement no A29-019-3. Male Wistar Rats were housed in the same place at 23°C, with a 12-h day light and fed ad libitum with standard rat pellets and had free access to water. Young male Wistar rats, from 5 to 15 days old, were used for this study.

### Cell Culture

The epidermal layer of the rat footpad was separated from the dermis by enzymatic digestion. Briefly, the tissue was incubated with dispase (15 U/mL, 37°C; Gibco, Paisley, UK) for 2 hours. Basal and suprabasal cells were dissociated from the epidermal layer by digestion with 0.05% trypsin-EDTA (Lonza, Walkersville, MD). The number of cells recovered and their viability were determined by haemocytometer counting using the trypan blue exclusion method. Cells were cultured in Dulbecco's Modified Eagle Medium (DMEM)/Ham's F12 Medium (Lonza, Walkersville, MD) supplemented with 5% fetal calf serum (FCS) (PAA Laboratories, Pasching, Austria) and Normocin™ (Invivogen, San Diego, CA) at 100 µg/mL as an antibiotic. The outer root sheaths of young rat whisker pads were dissected under a binocular microscope after a 1 hour of digestion with dispase (15 U/mL) and collagenase IV (100 U/mL) (Sigma, St. Louis, MO).

### Immunostaining

Tissues or cells were fixed for 10 to 30 min in 4% paraformaldehyde (pH 7.4), washed 3 times in PBS and incubated for 10 min in PBS containing 0.5% Triton X-100, followed by a 15-min incubation in PBS, 5% FCS and 0.1% Tween 20 at room temperature. Preparations were exposed to monoclonal mouse anti-cytokeratin 20 (CK20) antibody (1∶25; Progen GmbH, Heidelberg, Germany) overnight at 4°C. After rinsing, the preparations were exposed to polyclonal goat anti-mouse IgG conjugated to TRITC (1∶300; Sigma) diluted in PBS 0.1% Tween 20 and 1% FCS.

### Live-Cell Neurosecretory Granules Imaging

FM1–43 (3 mg/kg body weight) (Sigma) diluted in PBS was injected into rats intraperitoneally. Animals were sacrificed 24 hours later and skin biopsies were laid down on slides without fixation for analysis. Cells were stimulated by hypotonic solution or a TRPV4-agonist under an epifluorescence upright microscope (Olympus BX41). Pictures were taken with an Olympus C-5060 digital camera and analyzed with ImageJ software (http://rsbweb.nih.gov/ij). Only brightness and contrast were adjusted to improve analysis. The 3D surface plot plugin was used to confirm modifications of fluorescence intensity within cells. For quantification, 8-bits gray pictures were used. Background was subtracted. FM1–43-labeled MCs were circled using ROI (Region Of Interest) Manager and gray values were measured (n = 33 from 4 different experiments). Difference between values before and after 4αPDD exposure (1 µM) was analyzed using a Student's paired t-test.

### Vector Construction

Searches in rat for sequences similar to Math1 enhancers (GenBank: AF218258 [49]) were carried out using the BLAST program [50]. Two highly conserved domains, displaying 92 and 94% of homology to the sequences in human and separated by 80 nucleotides, were identified within a 1.51 kb sequence located 3 kb downstream of the Math1 coding region on chromosome 4q31. This sequence was amplified by PCR using the *Pfu* DNA polymerase (Promega, Madison, WI) and the following oligonucleotides, flanked respectively by EcoRI and XhoI sites: 5′ CGGAATTCCAAGGTCCAGCAATGAAGTTTGC 3′ and 5′ CGCTCGAGCCTCCCCTAGGCTTTGCTTGGC 3′. Thirty PCR cycles were performed with a denaturating temperature of 92°C for 30 sec, an annealing temperature of 60°C for 1 min and an amplification at 74°C for 2 min. The PCR product was purified, digested with EcoRI and XhoI and ligated into a pCMV-β-galactosidase vector that had been cut with the same restriction enzymes. In the resultant vector, pMath1-β-galactosidase, the CMV promoter was replaced by the Math1 enhancer.

### Transfection

The day before transfection, 1×105 epidermal cells or 20 whisker pads were seeded in 35 mm culture dishes containing medium supplemented with FCS without antibiotic. Six transfection reagents were tested. All reagents were used following the recommendations of the provider (Table 1). Briefly, (1) Cells were exposed for 30 min to 2 µg of plasmid and 500 µg/mL of DEAE-dextran (Sigma) in 200 µL of DMEM/F12. Subsequently, 700 µL of 80 µM chloroquine (Sigma) was added to the medium. A DMSO shock (DMSO 10% in DMEM/F12) was performed 3 hours later for 2 min and then the medium was changed. (2) Lipofectamine LTX (Invitrogen, Karlsruhe, Germany) was diluted in 200 µL of the DNA solution (2.5 µL of reagent per 1 µg of plasmid) and, after 30 min, the solution was added to the culture medium. (3) In the third trial, 10 µL of Lipofectine (Invitrogen) and 2 µg of plasmid were diluted each in 100 µL of medium. After 30 minutes, the two solutions were pooled and added to the culture medium 10 min later in a final volume of 1 mL. (4) Alternatively, 6.4 µL of Nanofectine® (PAA Laboratories, Pasching, Austria) and 2 µg of DNA were each diluted in 100 µL of medium. After 5 min, the solutions were pooled. After a 20-min incubation, the solution was added to the culture medium. (5) DNA (1 µg) was added to 4.4 µL of polyethyleneimine (PEI) (Sigma) and the mixture was diluted to a volume of 100 µL of DMEM/F12. The diluted mixture was incubated for 15 min prior to addition to the culture medium. (6) Twelve-μL of Transfast (Promega) and 2 µg of plasmid were each diluted in 100 µL of medium. After 5 min, the solutions were pooled and incubated for 10 min. The culture medium was then replaced by the DNA-Transfast solution. After 2 to 3 hours, 1 mL of DMEM/F12 was added to the cells.

**Table 1:** Transfection reagents used to transfect MCs.

Transfection Reagents	Amount used	plasmid	Final volume
DEAE-dextran	100 µg	2 µg	200 µL
Lipofectamine LTX	2.5 µL	1 µg	200 µL
Lipofectine	10 µL	2 µg	200 µL
Nanofectine	6.4 µL	2 µg	200 µL
Polyethylenimine	4.4 µL	1 µg	100 µL
Transfast	12 µL	2 µg	200 µL

Brief descriptions of the transfection protocols. Data were collected two days after transfection. Transfection reagents were evaluated by determining the number of blue cells seen in the presence of X-gal on the vibrissal outer root sheath.

### β-Galactosidase Staining

Two days after transfection, cellular β-galactosidase activity was assessed at 37°C using a reaction mixture composed of 1 mg/ml 5-bromo-4-chloro-3-indolyl-beta-D-galactopyranoside (X-gal), 4 mM K_3_Fe(CN)_6_, 4 mM K_4_Fe(CN)_6_ and 2 mM MgCl_2_ in DMEM/F12. All chemicals were purchased from Sigma. The first cells appeared blue within 30 min. Electrophysiological recordings were performed on the same day.

### Electrophysiological Analyses

The culture medium was replaced with an osmotic buffer (300 mosm) containing HEPES buffer (30 mM), NaCl (130 mM), KCl (3 mM), CaCl_2_ (4 mM) and glucose (10 mM) with the pH adjusted to 7.4 by NaOH addition. Transmembrane ionic currents were recorded from blue cells corresponding to MCs using a macro-patch clamp technique [51] that has been previously described [52]. Briefly, pipettes were pulled and heat polished from 1.5 mm diameter borosilicate glass (Clark Electromed, USA) with a DMZ-Universal puller (Zeitz Instruments, Germany). Resistance of the pipettes averaged 2 MΩ when filled with the recording pipette solution. Voltage pulses were delivered to the cells and current recordings were processed *via* a GeneClamp 500B amplifier and a CV-5-100U headstage (Axon Instruments) connected to a micro-computer through a 12-bit A-D/D-A interface (CED 1401+; Cambridge Electronic Design Ltd., UK). Voltage-clamp protocols and data acquisition were performed with WinWCP V3.2.5 (Whole Cell Program, J. Dempster, Strathclyde University, UK). Currents were low-pass filtered at 5 kHz and digitized at 48 kHz. We systematically checked Giga-seal and compensated for any observed leaky currents. Cells showing unstable seals were not used for recordings. The maximum conductance was calculated from the slope of the current to voltage relationship. TEA was used at 10 mM, ACh and RR were used at a final concentration of 10 µM. Histamine was used at a final concentration of 300 µM, Gadolinium (Gd^3+^) was used at 100 µM, and 4αPDD was used at 1 µM. All of these chemicals were supplied by Sigma. Suction was controlled by a pressure gauge. Mean values were compared by statistical tests (Student's t-test or Mann-Whitney as appropriate) after checking the normality of distribution. A significant difference was assumed for *p* values <0.05. Error bars show the standard error of the mean (SEM).
